# Home OCT Monitoring as a Safety Net for Early Detection of Recurrent Disease Activity in Neovascular Age-Related Macular Degeneration Under Standard Care

**DOI:** 10.3390/medicina62071241

**Published:** 2026-06-26

**Authors:** Deepak Sambhara, Ashkan M. Abbey, David A. Eichenbaum

**Affiliations:** 1 Eye Clinic of Wisconsin, Wausau, WI 54403, USA; 2 Texas Retina Associates, Dallas, TX 75231, USA; aabbey@texasretina.com; 3 Retina Vitreous Associated of Florida, St. Petersburg, FL 33711, USA; deichenbaum@rvaf.com

**Keywords:** AMD, neovascular age-related macular degeneration, vision loss, early detection, safety net, artificial intelligence, OCT, home OCT, telemedicine

## Abstract

*Background and Objectives*: Despite recent advancement, neovascular age-related macular degeneration (nAMD) remains a leading cause of irreversible vision loss. Undertreatment, fewer anti-VEGF injections and longer intervals than in clinical trials have been associated with sub-optimal visual outcomes. Visit-based regimens (Treat-and-Extend, PRN) may permit intervals of unrecognized retinal fluid between office visits. A home OCT system with near-daily self-imaging provides frequent structural retinal information between office visits that can support early detection of persistent or recurring fluid. The objective was to evaluate the duration and magnitude of fluid exposure between standard care visits and estimate the potential to shorten that exposure. *Materials andMethods*: Ad hoc analysis of three cohorts of treatment naïve and experienced nAMD eyes managed by standard care while participating in observational studies of the home OCT system, with treating physicians masked to home OCT data. AI-based analysis of fluid volume, rate of change and time of fluid onset was performed. *Results*: Data from 209 participants, mean age 76.4 years, 53% female, who performed 10,110 scans (6.0 scans/week) were analyzed. An amount of 119 eligible eyes provided data from 185 standard care intervals. Persistent or recurring fluid was identified in 121 (65%) intervals, on average 32 days prior to the next office visit. Of these, 84 (69%) had potential visit advancement within labeled minimal treatment intervals of 19 days. Mean fluid volume at the earliest possible notification was 26 nL and recurrence rate averaged 4.4 nL/day. *Conclusions*: A substantial proportion of patients experience unrecognized disease activity between visits. Home OCT monitoring provides adjunctive information to support early detection of fluid and may facilitate timely clinical evaluation. In this context, such monitoring may be considered reasonable and necessary to inform management of nAMD within established standards of care, while not replacing clinician-directed diagnosis or treatment decisions.

## 1. Introduction

Neovascular age-related macular degeneration (nAMD) remains a leading cause of irreversible vision loss among adults aged 50 years and older in developed countries, and its prevalence continues to grow as the population ages [1,2,3]. The introduction of intravitreal anti-vascular endothelial growth factor (anti-VEGF) therapy transformed the therapeutic landscape for nAMD, enabling the majority of patients in pivotal randomized controlled trials to maintain or gain visual acuity over the course of treatment [4,5,6]. Nevertheless, a persistent and well-documented gap exists between the visual outcomes achieved in pivotal clinical trials and those observed in real-world clinical practice [7,8].

Long-term observational studies have consistently demonstrated that visual acuity gains attenuate over time outside the controlled trial setting, with many patients experiencing progressive vision decline despite ongoing treatment [9,10,11]. A broad body of evidence suggests that the principal driver of this outcome gap is undertreatment, characterized by fewer anti-VEGF injections and longer treatment intervals than those employed in registrational trials [10,11,12]. Although preservation of vision is the ultimate goal, the main target biomarker driving efficient management is retinal fluid as observed on OCT [13,14]. Undertreatment can lead to prolonged exposure to retinal fluid [15]. Treat-and-extend (T&E) and pro re nata (PRN) regimens are primarily driven by the status of retinal fluid and have been widely adopted in an effort to reduce treatment burden on patients, caregivers, and clinical infrastructure; however, these approaches rely upon periodic in-office assessments to determine disease activity and guide dosing intervals [16,17].

A fundamental limitation of current disease management is the absence of a clinically integrated safety net to identify exudative reactivation between pre-scheduled office visits. By design, both the PRN and T&E approaches result on occasion in the arrival of patients to the pre-schedule visit with active exudation. When using PRN, no treatment will be administered until such exudation emerges, and when using T&E, during the extension process, it is estimated, based on limited available real-world data and the expected variability across clinics and physicians, that the majority of eyes eventually return with exudation, leading to shortening of the interval, with a variety of approaches on rechallenging the extension process [18,19]. Such disease activity may emerge days or even weeks before a patient’s appointment, and by the time reactivation is observed in clinic, the macula may already have been exposed to intraretinal or subretinal fluid for a prolonged interval of time. Cumulative fluid exposure and fluctuation have been associated with progressive loss of visual acuity and anatomical damage in eyes treated for nAMD, underscoring the clinical importance of timely detection and intervention [20,21,22].

Home-based optical coherence tomography (OCT) has emerged as a potential tool to bridge this surveillance gap by enabling frequent, patient-administered imaging between office visits [23,24]. While the feasibility, image quality, and patient adherence associated with home OCT have been described [24,25,26], the clinical implications of between-visit monitoring—particularly the frequency and magnitude of disease reactivation that occurs under standard care schedules—have not been comprehensively quantified.

The aim of this study was to quantify how often patients with nAMD develop disease reactivation prior to their next pre-scheduled office visit, using data obtained from observational trials of home OCT monitoring. Analyzing data from longitudinal studies in which the physicians were not privy to the home OCT output, therefore management was solely based on standard care methods, allows for the objective evaluation of the potential effect of the addition of a home OCT-based safety net in early detection of retinal fluid, including a distinction between intra- and sub-retinal compartments. The standard of care approach provides unbiased data, controlled within each patient, to the two possible alternative management paths, standard care vs. standard care supported by home OCT for earlier detection of fluid. We sought to characterize the timing and magnitude of between-visit recurrence and thereby inform the role of home monitoring in ongoing nAMD care.

## 2. Materials and Methods

We conducted an ad hoc analysis of data collected during three observational trials of a home OCT monitoring device [26,27,28]. The parent studies enrolled patients with a clinical diagnosis of nAMD in the study eye who were under active anti-VEGF therapy as part of their routine care. The studies were conducted in the US under IntegReview (Austin, TX, USA) (Approval Code: C2020.004, Approval date: 26 October 2020, ct.gov registration: NCT04650672, accessed 1 June 2026), Jaeb Center for Health Research (Tampa, FL, USA) (Approval Code: DRCRnet-AK, Approval date: 24 July 2022, NCT not available) and Advarra (Columbia, MD, USA) (Approval Code: C2021.001, Approval date: 13 April 2021, ct.gov registration: NCT04907409, accessed 1 June 2026). The source of the data of Cohort 2 is the DRCR Retina Network (Unique Federal Award Identification Number (FAIN) UG1EY014231 funded by the National Institutes of Health), but the analyses, content, and conclusions presented herein are solely the responsibility of the authors and have not been reviewed or approved by DRCR Retina Network.

Institutional Review Boards approvals, conducted in accordance with the tenets of the Declaration of Helsinki, and the participants signed an informed consent form. Enrolled patients were shipped a home OCT device (SCANLY Home OCT; Notal Vision Inc., Manassas, VA, USA) [29,30], and instructed to perform daily self-imaging of the study eye over monitoring periods ranging from 5 weeks to 6 months. The studies were observational, i.e., the home OCT scans were NOT available for physician review, and the management decisions were solely based on standard care office-based methods. All home OCT scans were transmitted to a centralized reading center, where the Notal OCT Analyzer (NOA) 1.0 (Notal Vision Inc., Manassas, VA, USA) analyzed each scan for the volume of hypo-reflective spaces typically representing retinal fluid and adjudicated for the timing of disease reactivation that was defined as HRS volume of > 3 nL reached prior to the patient’s next pre-scheduled office visit.

### 2.1. Study Population

For the present analysis, all eyes diagnosed with nAMD from three cohorts were included. It is a heterogeneous group that included naïve nAMD eyes recently diagnosed and treatment-experienced eyes that were routinely managed by US retinal specialists, hence generally representative of the US nAMD population undergoing standard care. Cohort 1 was collected from 15 participants of 3 months, at-home study of treatment-experienced eyes conducted between December 2020 and August 2021 [24]. Cohort 2 was collected from 14 participants of a 6-month study of treatment-naive eyes conducted between September 2021 and November 2022 [26]. Cohort 3 was collected from 180 participants of at-home OCT pivotal study, conducted between June 2021 and December 2022 and included varying intervals, starting with the previous injection prior to study enrollment followed by a 5-weeks home OCT monitoring period at the end of the interval, hence providing the relevant data for the current analysis [28]. Since the home OCT monitoring period was limited to 5 weeks, the results represent an underestimation of the full potential of early detection of recurrence.

### 2.2. Outcome Measures

1. The duration of standard care monitoring intervals.
2. The duration within each interval from the initial identification of recurring or persistent fluid to the end of the interval. Of note, fluid that was observed at the beginning of an interval and resolved during the interval was not included in the analysis.
3. The shortening potential of each interval considering the 21-days blackout period while considering that the minimal allowed interval between treatments per the drugs’ label is 28 ± 7 days [31,32]. The “blackout” period of twenty-one days from the previous treatment was selected as the minimal allowed interval that can issue the earliest notification to the physician and allow time for scheduling of an office visit.
4. The volume of hypo-reflective spaces associated with retinal fluid as calculated by NOA at the earliest time point that would allow a notification to the physician following the blackout period.
5. The proportion of intervals in which review of the output of the home OCT monitoring showed the presence of retinal fluid, whether persistent or recurring, that could have prompted an unscheduled office visit prior to the next routine appointment.
6. The proportion of intervals in which the standard care interval was longer than 21 days, and the earlier identification of fluid could have informed a decision to shorten the interval and bring the patient earlier to an office visit in order to minimize fluid exposure.
7. The distribution of number of days prior to the scheduled follow-up visit at which retinal fluid was first detected on home OCT.
8. The distribution of number of potential shortenings to the intervals.
9. The average daily rate of fluid accumulation following initial detection of reactivation, expressed in nanoliters per day (nL/day).

**Figure 1:**
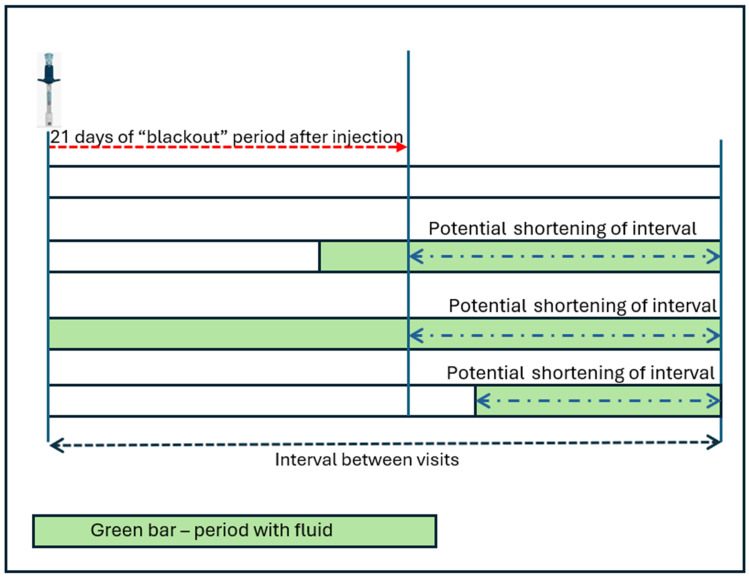
A graphical explanation of the elements of the analysis.

Figure 1 includes a graphical explanation of the elements of the analysis. Descriptive statistics were used to characterize baseline demographics and testing behavior, including age, gender, total number of home OCT scans performed, and average scan frequency. Continuous outcomes are reported as mean ± standard deviation (SD), median, min, max and categorical outcomes are reported as counts with percentages.

## 3. Results

The demographics and baseline characteristics are included in Table 1.

The data from the three cohorts allowed us to analyze 185 intervals between std. care office visits, with a weighted mean (SD) duration of 53 (21.6) days. During 121 (65%) of the intervals, retinal fluid, persistent or recurring, was present till the end of the interval. The weighted mean (SD) duration of fluid exposure during the intervals with observed fluid was 32 (13.1) days. These findings represent early detection of retinal fluid prior to a standard care visit.

**Figure 2:**
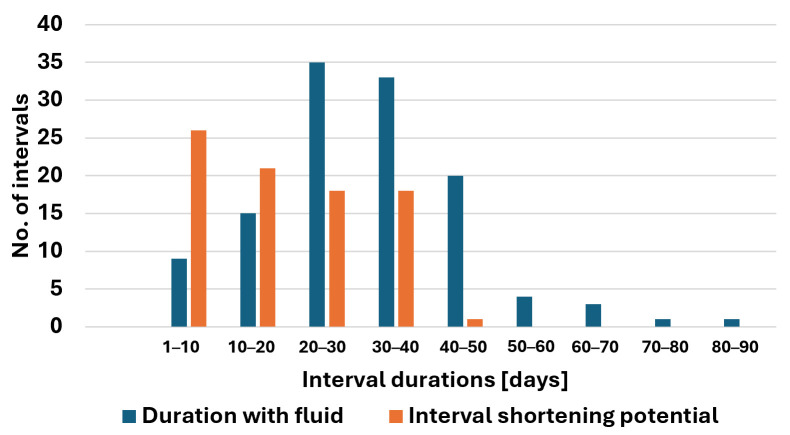
Frequency distribution of duration with fluid and periods with fluid exposure shortening.

For the purpose of evaluating the effect of early detection on the management of eyes with nAMD with current therapy, e.g., faricimab and aflibercept 8 mg, it is assumed that treatment may be administered not more frequently than approximately every 28 days +/− 7 days. Of the 121 intervals with fluid reaching the end of the interval, 84 (69%) had fluid on day 21 after the prior visit or later. The weighted mean (SD) duration of presence of fluid outside of the 21-days “black-out” period was 19 (10.0) days. This difference between the observed intervals and the potentially shorter interval due to early detection of retinal fluid with the home OCT and considering the minimal retreatment interval per the drug label was clinically and statistically significant with *p*-values < 0.00001 for all cohorts. The frequency distribution of the intervals and the potential shortening are included in Figure 2.

The weighted mean of the volume of retinal fluid detected upon first opportunity for notification following the blackout period was 26 nL.

**Figure 3:**
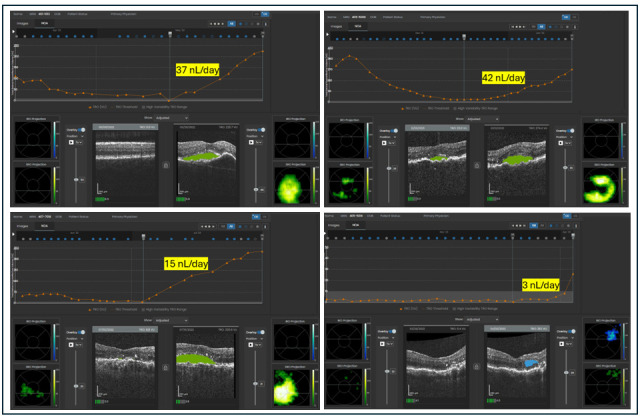
Examples of fluid volume trajectories with rapid increase.

Following initial detection, fluid accumulated at an average rate of 4.4 ± 7.7 and a range of 0.4–42 nL/day. Figure 3 depicts four cases of rapid fluid accumulation.

Details of the analysis by cohort are presented in Table 2.

## 4. Discussion

Studies of home OCT monitoring typically include review of the self-imaging results by the managing physician. Earlier studies of the system, prior to its FDA marketing authorization, in which the testing results were not available to the physician, provided a unique opportunity to objectively evaluate the system’s efficacy as a safety net between standard care visits. This approach may be compared to the methodology used in the first pivotal anti-VEGF MARINA study [4], in which the control arm received only sham injections, an approach that has not been repeated since.

In the current analysis of home OCT trials data, a meaningful proportion of close to two-thirds of the patients with nAMD presented with persistent fluid or disease reactivation prior to their next scheduled office visit, while eyes without fluid or fluid that resolved prior to the next visit were excluded. These reactivations were detected on average 32 days and maximum 61 days before the patient’s scheduled appointment, indicating that many eyes were exposed to fluid for periods longer than the average. Left unmonitored, this represents a period of potential structural and functional harm that is not addressed by current standard-of-care follow-up schedules. The analysis conservatively self-imposed a “blackout” period of 21 days per the drug’s label. Eyes that had persistent fluid, or newly emerging fluid during that period, could be considered for even earlier off-label treatment. However, when complying with the mandatory waiting time between injections, 45% of all eyes, which was 69% of eyes with persistent or recurring fluid during the interval, had the potential to shorten the wait for office visit and potential treatment by a weighted average duration of 19 days (with apparent variability among the three cohorts of 17, 13, and 26 days). For reference, this is more than the typical two-week extension or shortening step in a T&E regiment. This exposure to fluid may be considered clinically significant, especially for eyes on the longer side of the distribution. NOA quantification showed that the weighted average fluid volume upon earliest possible notification was 26 nL, far exceeding a typical 10 nL notification threshold used in studies of home OCT-guided disease management [33], emphasizing the possibly harming delay in care of these eyes. With the observed minimal fluid volume of 3.2 nL at the beginning of the interval relevant for notifications and significant rise in fluid until the subsequent office visit, there is little concern for false identification of fluid that may skew these findings. On the other hand, the maximal advancement in possible time to notification under the “blackout” limitation was 41 days, the maximal fluid volume was 40 nL and the maximal fluid increase rate was 41 nL/day, indicating potential associations with retinal damage or negative long-term visual prognosis. For eyes with recurring fluid, the weighted mean rate of fluid accumulation was 4.4 nL/day, indicating the acute nature of the rapid disease process and the potential benefit of near-daily, at-home self-imaging—a dynamic not captured even with biweekly in-office imaging used clinically in some cases. The cases in Figure 3 represent recurrence episodes that would have most likely resulted in prompt visits to the clinic, followed by earlier treatment, if this data would have been available as part of clinical care.

These findings show that a substantial number of patients are effectively undertreated under prevailing treatment regimens not because of clinician oversight, but because conventional in-office follow-up intervals lack the temporal resolution to identify early reactivation. As an example, a strict T&E study reported that 65% of patients demonstrated recurrent exudation upon interval extension [18]. We hypothesize that this pattern reflects a fundamental tension in the management of nAMD: retina specialists appropriately seek to minimize the burden of frequent office visits and injections on patients and caregivers yet have historically lacked objective tools to predict or detect disease recurrence between visits. Consequently, treatment intervals are often extended empirically, with disease activity emerging as a lagging indicator of undertreatment rather than a leading indicator of need. The quantitative characterization of cumulative between-visit fluid exposure provided by the current analysis offers direct evidence of the clinical manifestation of this surveillance gap. To benefit from the early detection of reactivation, home OCT monitoring requires office visits to be scheduled, and patient transportation be arranged on short notice.

The results of this analysis support the clinical utility of home OCT monitoring as an adjunctive safety net to the standard of care. By enabling early detection of reactivation days before a pre-scheduled visit, home OCT may permit timely office visits and intervention, reduce cumulative fluid exposure and therefore support the primary objective of management of nAMD to potentially preserve long-term visual outcomes [20,21,34,35]. The observed rate of daily fluid accumulation further supports the notion that early detection is not merely convenient but mechanistically relevant: even modest delays in recognition may translate into measurable increases in fluid burden on single occasions and over the course of long-term disease management.

As evidenced by the high adherence to near-daily self-imaging six times per week on average, the high frequency of monitoring does not put an undue burden on patients. Access to a device in the comfort of their home has the potential to enable elderly nAMD patients to actively participate in managing this chronic, sight-threatening disease over the long term.

The marketing authorization by the FDA and the experience gained from this report and other published results support the operationalization of management of nAMD by retinal physicians, including setting up efficient workflows for reviewing reports, setting notification thresholds, responding to alerts and accommodating home OCT-driven visits.

The findings of this analysis gain further importance with the advent of long-acting treatment solutions. Treatment interval extensions in steps of up to 4 weeks up to durations of 24 weeks in case of intravitreal injections may further exacerbate the risk of undetected early fluid recurrence. Realization of the full durability potential of such treatment solutions in routine care may require synergistic implementation of remote patient monitoring solutions as recently discussed [36].

Several limitations of this analysis warrant consideration. This was an ad hoc analysis of data collected in the setting of trials, and patient behavior, adherence, and imaging frequency may not fully reflect what would be observed in unselected real-world populations. The monitoring periods were between 5 and 24 weeks. Long-term outcomes of active management with home OCT, including adherence to expected years of frequent self-imaging, as well as the impact on visual acuity and anatomic stability, were not assessed within the scope of this analysis. Cohort 3 was monitored with HOCT for only 5 weeks causing an underestimate of the gain in earlier detection of fluid. The clinical and visual significance of the fluid accumulations detected in this study are not known.

## 5. Conclusions

A significant proportion of patients with nAMD experience unrecognized disease activity and cumulative retinal fluid exposure prior to their next pre-scheduled office visit. These findings highlight the limitations of visit-based monitoring and suggest that a substantial fraction of real-world undertreatment is a direct consequence of the absence of between-visit surveillance tools. Home OCT monitoring provides a potentially clinically meaningful addition to the management of nAMD, with the ability to reduce undertreatment, mitigate avoidable fluid exposure, and potentially ultimately improve long-term visual outcomes for patients with this sight-threatening disease.

## Figures and Tables

**Table 1:** Demographics and baseline characteristics.

	Cohort 1	Cohort 2	Cohort 3	Total/Weighted Mean
No. of participants ^1^	15	14	180	209
No. of eyes with nAMD	24	14	180	218
Age,	* 73.4 ± 6.5	** 74 (69, 83)	* 77.1 ± 7.2	76.5
Gender, % female	53%	43%	57%	53%
BCVA	* 20/40 (20/20–20/200)	** 20/63 (20/32, 20/200)	* 20/40 (20/20–20/320)	-
Prior injections, mean (SD)	33 ± 28	0	26.4 ± 26.5	-
Total no. of self-tests	2380	2304	5426	10,110
Weekly frequency of test, mean (SD)	5.7 ± 0.9	6.3 (0.6)	6.02 (NA)	6.0
Mean duration of monitoring with the home OCT [weeks]	12	24	5	-

^1^ Not all participants had eligible intervals. * mean (SD). ** median (IQR).

**Table 2:** Analysis of intervals between standard care visits by cohort.

	Cohort 1	Cohort 2	Cohort 3 ^1^	Total/Weighted Mean
No. of eyes diagnosed with nAMD	21	14	84	119
No. (%) of intervals	38 (100%)	63 (100%)	84 (100%)	185
Duration of intervals [days]				
Mean (SD)	47.4 (19.6)	40.3 (11.9)	65.0 (29.7)	53 (21.6)
Median	41.5	42	56.5	48
Min	15	28	33	28
Max	100	84	189	135
No. (%) of intervals with persistence or emerging fluid that is present at end of interval	30 (78.9%)	51 (81.0%)	40 (47.6%)	121 (65%)
Duration with fluid within intervals [days]				
Mean (SD)	38.4 (19.4)	31.9 (13.1)	26.3 (8.4)	32 (13.1)
Median	35	28	29.5	30
Min	4	1	5	3
Max	87	64	38	61
No. (%) of intervals with persistence or emerging fluid that is present at end of interval that was observed >21 days from prior visit and assumed injection	20 (66.7%)	34 (66.7%)	30 (75.0%)	84 (69.4%)
Duration with fluid after blackout period of 21 days till end of interval—potential interval shortening				
Mean (SD), *p*-value ^2^	17.2 (10.0), *p* < 0.00001	13.8(10.8), *p* < 0.00001	25.7 (9.2), *p* < 0.00001	18.9 (10.0)
Median	13.5	8	29	16.8
Min	4.0	1	5	3.1
Max	39.0	46	38	41.5
Fluid volume at time of earliest notification after 21-days blackout period [nL]				
Mean (SD)	40.1 (42.2)	17.2 (16.9)	25.3 (51.5)	26.0 (35.3)
Median	19.4	8.7	7.5	10.8
Min	3.6	3.1	3.1	3.2
Max	115.8	72.2	231.9	139.6
Fluid accumulation rate during recurrence [nL/day]				
Mean (SD)	3.4 (2.2)	1.9 (2.0)	6.0 (10.1)	4.4 (7.7)
Median	3.1	1.3	1.9	1.8
Min	0.5	0.4	0.4	0.4
Max	8.9	11.0	41.5	41.5

^1^ Excluded eyes that were enrolled with known fluid that still allowed a short delay and were treated few days after initiating home OCT monitoring in the Week 1 visit. ^2^ Paired *t*-test of the difference between the original interval and the potentially shorter interval in intervals that were >21 days.

## Data Availability

The relevant data presented in this study are available on request from the corresponding author.

## Abbreviations

The following abbreviations are used in this manuscript:

AI	Artificial Intelligence
AMD	Age-related Macular Degeneration
DRCR	Diabetic Retinopathy Clinical Research
FDA	Food and Drug Administration
HRS	Hypo Reflective Spaces
nL	Nano Liter
NOA	Notal OCT Analyzer
PRN	Pro re nata
SD	Standard Deviation
T&E	Treat and Extend
VEGF	Vascular Endothelial Growth Factors
